# Some Groups of Diarrhœic Symptoms

**Published:** 1883-04

**Authors:** B. F. Joslin


### SOME GROUPS OF DIARRHŒIC SYMPTOMS.

B. F. Joslin, M. D.*

These, being Nature's groupings, are of more practical value than those which are formed merely from the *Materia Medica*, without reference to any observation of their actual occurrence in natural disease. The author, like most practitioners, has observed all of them in the latter, and most of them a great number of times. Those who practice accurately, often study to find the remedies adapted to groups; if they recorded and preserved the results of their calculations, the aggregate of those collected by different physicians would form a valuable repertory for general practice.

Acrid and brown stools: Ars., verat.

Black and green stools: *Ars.*, ipec., merc., phos., sulph.-ac., *verat.*

Blackish stools after abuse of ipecac: Chin.

Bloody, mucous, and fœtid stools: Lach., *merc-c.*, sulph., sulph-ac.

Brown and green stools: *Ars.*, dulc., magn., magn-m., merc-c., *sulph.*, verat.

Brown and watery stools: *Ars.*, dulc., sulph., tart.

Brown stools, with nausea from movement: Ars.

Clay-colored and frothy stools in diarrhœa: Calc., rhus-rad.

Diarrhœa after fruit; with sighing respiration: Lach.

— at night, with distention of the stomach and abdomen after meals: Bor., bry., caust., *cham.*, *chin.*, dulc., *kali.*, lach., *merc.*, *puls.*, *rhus-tox.*, *sulph.*

— during dentition; white coat on the tongue; yellowish stools: Calc., ipec., *merc.*, *sulph.*

— painless and at night: *Ars.*, bor., bry., canth., *cham.*, *chin.*, dulc., *merc.*, *puls.*, *rhus-tox.*, *sulph.*, verat.

— with colic and at night: *Ars.*, bor., bry., cham., dulc., lach., *merc.*, puls., *rhus-tox.*, *sulph.*, verat.

— with colic and tenderness of the abdomen: Acon., *canth.*, *cham.*, *merc-c.*, *nux*, *puls.*, *rhus-rad.*, stram., *sulph.*, tereb., *verat.*

— with colic; stools fœtid: *Ars.*, bry., coloc., ipec., lach., *merc.*, nux, stram., *sulph.*

— with colic; stools green: *Ars.*, bor., coloc., phos., puls., *verat.*

— with white coat on the tongue, and yellow stools: Amb., calc., ign., ipec., *merc.*, oleand., petr., phos., puls., *sulph.*

---

* Taken from the work on cholera, etc., of the late Dr. B. F. Joslin.

Diarrhœa with involuntary evacuations at night: *Ars.*, *bry.*, *chin.*, lach., *merc.*, puls., *rhus-tox.*, *sulph.*, verat.

— with sweat on the face, nausea and stiffness of the neck, and pain in it when moving it: *Camph.*

— fœtid and green stools: *Ars.*, cham., coloc., lach., *merc.*, merc-c., nux, sep., *sulph.*, sulph-ac., tab.

— fœtid stools, in diarrhœa with colic: *Ars.*, bry., coloc., ipec., lach., *merc.*, nux, stram., *sulph.*

— frothy and involuntary stools, in diarrhœa: *Chin.*, merc., op., *rhus-tox.*, *sulph.*

— green and slimy stools: *Ars.*, *bell.*, bor., canth., cham., coloc., dulc., ipec., laur., *merc.*, *nux*, phos., puls., sep., staun., *sulph.*, sulph-ac., tab.

— green, slimy, and undigested stools: *Ars.*, bor., cham., nitr-ac., *phos.*, *phos-ac.*, rheum, *sulph.*, sulph-ac.

— green, sour, and undigested stools: *Merc.*, *sulph.*

— watery stools, in diarrhœa with colic: *Ars.*, *cham.*, dulc., *lach.*, *nux*, puls., *rhus-tox.*, *sulph.*, *k.-bi.*

— watery stools, with brown coat on the tongue, and vomiting at night: Bell., *phos.*, sulph.

